# Influence of the Region of Injury on Risk of Mortality in Severely Injured Patients Stratified by Age: An Analysis of 98,481 Patients from the TraumaRegister DGU^®^

**DOI:** 10.3390/jcm15114147

**Published:** 2026-05-27

**Authors:** Jonin Serafin Zumsteg, Yannik Kalbas, Lara Zankena, Franziska Ziegenhain, Julian Scherer, Nicolas Eibinger, Rolf Lefering, Hans-Christoph Pape, Kai Oliver Jensen, the TraumaRegister DGU

**Affiliations:** 1 Department of Trauma, University Hospital Zurich, 8091 Zurich, Switzerland; yannik.kalbas@usz.ch (Y.K.); franziska.ziegenhain@usz.ch (F.Z.); julian.scherer@usz.ch (J.S.); hans-christoph.pape@usz.ch (H.-C.P.);; 2 Department of General, Visceral and Vascular Surgery, Cantonal Hospital Baden, 5404 Baden, Switzerland; lara.zankena@balgrist.ch; 3 Department of Orthopaedics and Trauma, Winterthur Cantonal Hospital, 8401 Winterthur, Switzerland; 4 Center for Musculoskeletal Surgery, Charité Universitätsmedizin Berlin, 10117 Berlin, Germany; 5 General and Trauma Surgery, Uster Hospital, 8610 Uster, Switzerland; 6 Division of Trauma Surgery, Department of Orthopaedics and Trauma, Medical University of Graz, 8036 Graz, Austria; nicolas.eibinger@medunigraz.at; 7 Institute for Research in Operative Medicine (IFOM), University of Witten/Herdecke, 51109 Cologne, Germany; rolf.lefering@uni-wh.de; 8 Department of Trauma and Orthopedics, Hospital Bülach AG, 8180 Bülach, Switzerland; 9 Committee on Emergency Medicine, Intensive Care and Trauma Management (Sektion NIS) of the German Trauma Society (DGU), 10623 Berlin, Germany; support@auc-online.de

**Keywords:** severely injured patients, epidemiology, trauma registry, mortality, head injury, trauma centers, retrospective cohort study, trauma mechanism, geriatric trauma

## Abstract

**Background/Objectives**: The growing elderly population and concomitant increase in physical activity of older adults has led to a growing number of seriously injured elderly patients. The aim of this retrospective cohort study was to investigate the influence of the leading region of injury in severely injured patients on the risk of mortality in different age groups, with focus on elderly patients. **Methods**: Data from the TraumaRegister DGU^®^ from 2015 to 2020 were analyzed, including severely injured patients admitted to Swiss, German and Austrian trauma centers. Inclusion criteria were a minimum age of 18 years and an Abbreviated Injury Scale (AIS) score of three or higher in at least one of the body regions. Descriptive analysis and odds ratios for mortality derived from multivariable analysis were calculated, stratified by age and leading region of injury. **Results**: Out of 213,216 patients, 98,481 met the inclusion criteria. Mortality increased from 6.9% in the control group (18–54 years) to a maximum of 35.9% in the 90+ age group. Leading head injuries had a mortality rate of 22%. The odds ratio for the risk of mortality increased with age and reached a maximum value of 17.0 in the 90+ age group. However, the increase in risk of mortality for leading head injury with increasing age was lower than in the other regions, with an OR of 11.7 in the 90+ age group. In contrast, the group with a leading thoracic injury increased to an OR of 22.5, abdomen to an OR of 75.2 and extremities to an OR of 28.7. **Conclusions**: The risk of mortality from traumatic head injury is less pronounced in elderly people compared to other injury regions. Our data suggests that traditional scoring systems like the AIS might not display nuances of different injury severities in different age groups, especially for head injuries caused by low-energy trauma and therefore should be reevaluated.

## 1. Introduction

The proportion of people over 60 years of age is steadily increasing [1]. The age structure of the Swiss population has changed substantially during the 20th century [2]. The percentage of young people (under 20 years of age) decreased from 40.7% (1900) to 20.0% (2019), whereas the population of people over 64 years of age increased from 5.8% to 18.7%, and people over the age of 80 years increased from 0.5% to 5.3% [2]. This demographic aging process of, e.g., the Swiss population, is a consequence of increasing life expectancy and declining birth rates. By 2050, the proportion of people over 65 years is expected to rise from 18.7% (2019) to approximately 25.6% [2]. Over the past 20 years, the percentage of people aged 65 and over has increased in all European Union member states [3]. Accordingly, this and the higher activity and mobility in elderly people are considered to be the reasons for the increase in the number of severely injured older patients [4,5].

It is well known that the pattern of injury and the mechanism of injury differ at different stages of life and it has been shown that mortality changes with different anatomical regions involved [5,6,7,8]. The literature shows that older severely injured patients require different care than younger ones [9,10,11]. In addition, it is also shown that age is associated with higher frailty which leads to a more adverse outcomes [12,13].

Based on the anatomical changes and different accident mechanisms, we hypothesize that the risk of death for traumatic head injuries in severely injured patients increases less with age than for other leading injuries. Thus, the aim of this study was to investigate the influence of different injury regions and injury severity on the risk of mortality in different age groups.

## 2. Materials and Methods

### 2.1. The TraumaRegister DGU^®^

The TraumaRegister DGU^®^ (TR-DGU) of the German Trauma Society (Deutsche Gesellschaft für Unfallchirurgie, DGU) was founded in 1993. The aim of this multicenter database is a pseudonymized and standardized documentation of severely injured patients.

Data are collected prospectively in four consecutive time phases from the site of the accident until discharge from hospital: (A) pre-hospital phase, (B) emergency room and initial surgery, (C) intensive care unit (ICU) and (D) discharge. The documentation includes detailed information on demographics, injury pattern, comorbidities, pre- and in-hospital management, course in intensive care unit, relevant laboratory findings including data on transfusion and outcome of each individual. The inclusion criterion include admission to hospital via emergency room with subsequent ICU/ICM care or reaching the hospital with vital signs and death before admission to the ICU. The infrastructure for documentation, data management, and data analysis is provided by AUC—Academy for Trauma Surgery (AUC—Akademie der Unfallchirurgie GmbH), a company affiliated with the German Trauma Society. The scientific leadership is provided by the Committee on Emergency Medicine, Intensive Care and Trauma Management (Sektion NIS) of the German Trauma Society. The participating hospitals submit their data pseudonymized into a central database via a web-based application. Scientific data analysis is approved according to a peer review procedure laid down in the publication guideline of the TraumaRegister DGU^®^.

The participating hospitals are primarily located in Germany (90%), but a rising number of hospitals of other countries contribute data as well (at the moment from Austria, Belgium, China, Finland, Luxembourg, Slovenia, Switzerland, The Netherlands, and the United Arab Emirates). Currently, approx. 31,000 cases from about 700 hospitals are entered into the database per year.

Participation in the TraumaRegister DGU^®^ is voluntary. For hospitals associated with TraumaNetzwerk DGU^®^, however, the entry of at least a basic dataset is obligatory for reasons of quality assurance [14].

### 2.2. Patients

This study is a retrospective cohort study covering the period from 1 January 2015 to 31 December 2020. The reporting of data in this study is in accordance with the STROBE (Strengthening the Reporting of Observational Studies in Epidemiology) guidelines [15].

Patient data from the TR-DGU were analyzed. The study was restricted to Swiss, German and Austrian trauma centers reporting to the TR-DGU. We analyzed severely injured patients meeting the following inclusion criteria: Primary admission to the hospital via the resuscitation room with subsequent need for intensive care or arrival at the hospital with vital signs and death in the hospital before admission to the intensive care unit. In addition, at least one of the body regions must have had an injury severity score of 3 points or more according to the Abbreviated Injury Scale (AIS) [16]. Only patients who were at least 18 years at admission were evaluated. We excluded patients who were early (<48 h) transferred out and patients with a prior documented limitation of treatment and death within 48 h of hospital admission.

Patients were stratified by age group: Control group aged 18–54 years; patients over 55 years were divided into subgroups with 5-year increments. Patients who were 90 years or older were grouped together. We further divided the patients into several subgroups based on the most severe region of injury (head, thorax, abdomen, extremities, and multiple leading body regions). A leading region was defined as an injury in the anatomical region with a Score of the Abbreviated Injury Scale (AIS) ≥ 3 (AIS Version 2005 Update 2008).

To be categorized as one of the above, any additional injury in another anatomical region must have a maximum AIS of 2. If injuries in multiple regions had an AIS of 3 or more, they were included in the more than one leading injury category.

### 2.3. Statistical Analysis

Descriptive analysis was performed with mean and standard deviation (SD) for continuous data and number of patients with percentages for categorical variables. In case of seriously skewed data, medians with quartiles were reported rather than mean/SD. Given the large number of cases in both study groups, even small differences (e.g., 1% difference in categorical data) would reach formal statistical significance. Therefore, formal significance testing was not conducted in order to avoid ‘significant’ results in case of minor differences. Differences in clinical relevance are certainly also statistically significant with more than 40,000 cases per group.

The primary endpoint of the study was hospital mortality. In order to analyze the risk of death in different age groups, a logistic regression was performed and the risk of death in elderly patients was expressed as an odds ratio relative to the group of 18–54-year-olds (reference group). Further predictors such as sex, New ISS [17], GCS ≤ 8, blood transfusion, coagulopathy and pre-existing ASA (American Society of Anesthesiologists) score of 3/4 were analyzed in the logistic regression.

### 2.4. Ethical Consideration

The present study is in line with the publication guidelines of the TraumaRegister DGU^®^ and registered as TR-DGU project ID 2021-023. The research conducted in this study adheres to the ethical principles outlined in the 1964 Declaration of Helsinki and its subsequent revisions. The data used for this study originates from an external quality assessment initiative, utilizing routinely accessible data. Collecting this data does not necessitate an additional ethical approval. For the purposes of scientific analysis and publication, the data has been condensed, omitting specific details such as the date and time of injury, as well as the identifying information of the treating hospital.

During the preparation of this manuscript/study, the authors used ChatGPT (OpenAI, GPT-5.5) for the purposes of translations and linguistic corrections. The authors have reviewed and edited the output and take full responsibility for the content of this publication.

## 3. Results

**Figure 1:**
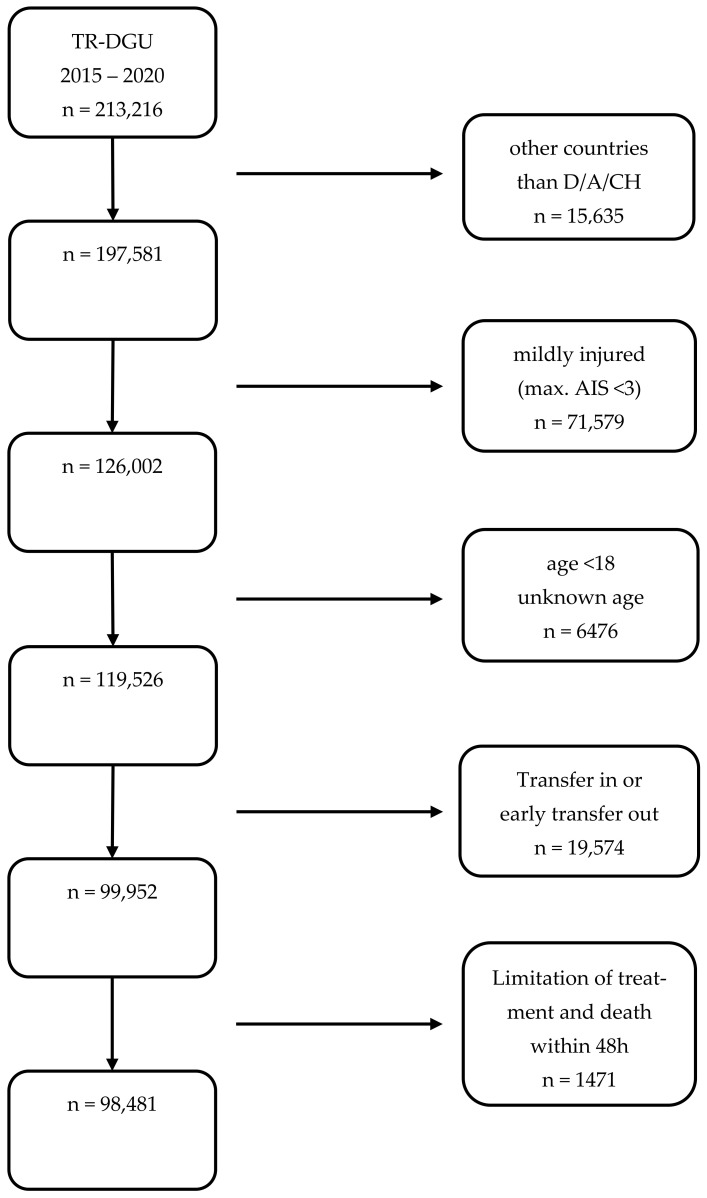
Selection process TR-DGU: TraumaRegister DGU^®^, D: Germany, A: Austria, CH: Switzerland, AIS: Abbreviated Injury Scale, n: number of patients.

During the analyzed period, 213,216 patient datasets entered the registry. After exclusion of countries other than Germany, Austria or Switzerland (D/A/CH), patients with minor injuries (maximum AIS < 3) and patients with unknown age or those younger than 18 years old, 119,526 were left. Then, 19,574 were excluded due to referral or early transfer out, and 1471 due to limitation of treatment. In total, 98,481 patients treated in 724 hospitals fulfilled the inclusion criteria and constitute the study population. The selection process is shown in Figure 1.

Table 1 shows the epidemiological data of the included patients. The control group, defined as patients aged 18 to 54 years, consisted of 44,844 patients (45.5%). Further subdivisions were carried out in 5-year increments, up to the age group ≥ 90 years with 2717 patients (2.8%).

We found a leading head trauma in 22.0% (n = 21,653) of the study population, whereas thorax trauma made up 24.2% (n = 23,795), abdominal trauma 2.5% (2442) and extremity trauma 15.4% (n = 15,161) In total, 36.0% (n = 35,430) reflected the group with more than one leading body region injury. The distribution between the control group and the elderly patients is shown in Table 1.

**Figure 2:**
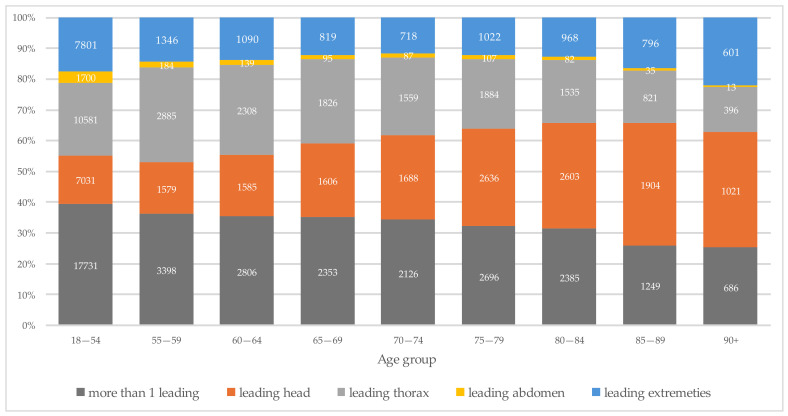
Relative distribution of injury patterns across age groups, including the absolute number of cases in each group.

Injury patterns changed markedly with age, particularly the group with multiple body regions decreasing from 39.5% in the control group to 25.2% in those over 90 years of age. Further, leading thorax trauma also decreased from 23.6% to 14.6% and abdominal trauma from 3.8% to 0.5%. In contrast, leading head injuries increased from 15.7% in the control group to 39.6% in the 85–89-year-old group. Leading extremity injuries had the lowest prevalence in the age group of 70–74 years with 11.6%. In the control group it is 17.4% and in the age over 90 group it is the highest with 22.1% (Figure 2).

The most prevalent trauma mechanism assessed was low falls (height < 3 m) in 26,190 patients (26.8%), followed by car accidents in 18,665 (19.1%), high falls (height > 3 m) in 15,242 (15.6%), motorcycle accidents in 12,362 (12.7%), bicycle accidents in 9936 (10.2%) and pedestrian accidents in 5233 (5.4%) (Table 2). The elderly group experienced substantially more falls from low heights, accounting for approximately 40% of cases, compared with 11% in the control group. Conversely, the control group experienced substantially more car accidents, representing 25% of cases versus 14% in the elderly group, and more motorcycle accidents, representing 18% versus 8%.

The overall mean in-hospital mortality was 12.9% and increased from 6.9% in the control group to 35.9% in the 90+-year-old group. The mortality for leading head injury was 22.6%, multiple leading injuries 17.2%, leading abdominal injury 6.1%, leading thorax injury 4.3%, and leading extremity injury 3.5% (Table 3).

**Figure 3:**
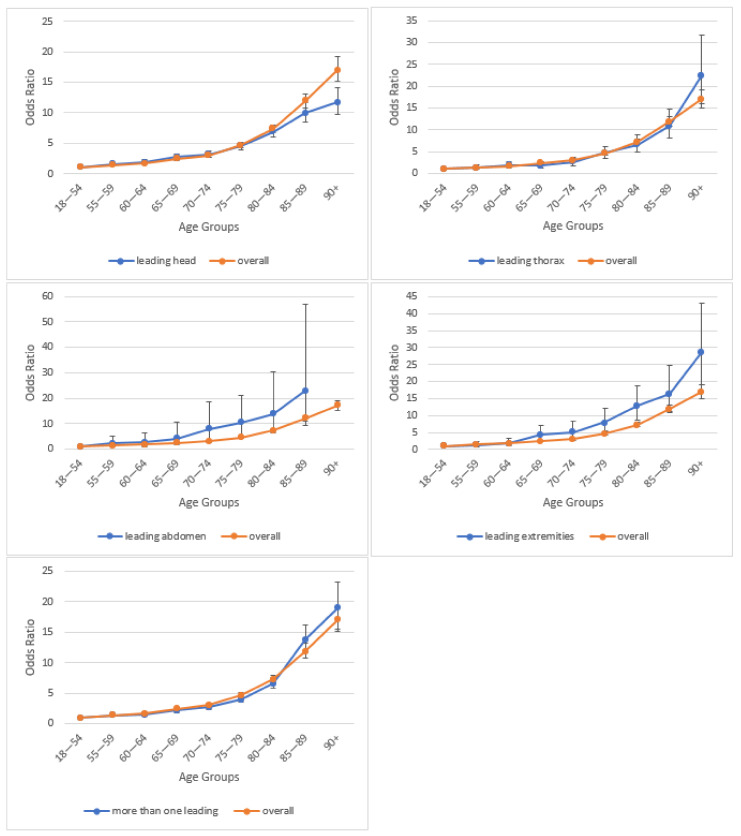
Risk of death in increasing age groups, expressed as adjusted odds ratios relative to the control group (age 18–54). The analysis of all cases (overall) was repeated in subgroups according to the leading body region. Adjusted odds ratios are presented together with their 95% confidence intervals.

The increase in the odds ratio for mortality with age stratified by subgroup is shown in Figure 3. The odds ratio amongst all assessed patients increased from one in the control group to 17.0 in the group older than 90 years. In the group with head injury as the leading injury, the odds ratio increased to 11.7 in the group over 90 years old, to 22.5 in the group with leading thorax injury, to 28.7 in the group with leading extremity injury, and to 19.0 in patients with more than one leading injury.

For patients with leading abdominal injury, the odds ratio would increase to 75.2 in patients older than 90 years. However, this estimate was associated with a very wide confidence interval due to the low number of cases in this subgroup (n = 13) and was therefore omitted from the figure. Nevertheless, a marked increase was already observed in the 85–89 years age group, with an odds ratio of 22.9.

The further predictors for mortality for the overall population and the individual leading injury regions are shown in Table 4.

## 4. Discussion

The aim of the present study was to investigate how different anatomical locations of injury amongst different age groups affect mortality. We analyzed data from 98,481 patients included in the TR-DGU. Several differences in trauma mechanism, injury pattern, mortality and risk of mortality were identified. Our main results include the following:

- With increasing age, there was a reduced increase in risk of mortality in the group with leading injury to the head compared to other leading anatomical regions.
- We observed a increase in overall mortality with age.
- We observed a difference in the leading injury between young and old patients.

### 4.1. Injury Patterns

Our analysis was able to show a marked difference in injury patterns between younger and elderly patients. Specifically, the portion of leading head injuries increased steadily with age, whereas the rate of leading abdominal, thorax, and more than one leading injury group decreased with age. This might be due to different trauma mechanisms depending on age group since the younger population more frequently engages in more high-risk activities compared to elderly individuals [18]. Brown et al. showed a similar trend where simple falls led to more head injuries in elderly patients [7].

### 4.2. Mortality

The in-hospital mortality amongst all assessed patients was 12.9%. The data presented demonstrated an increase in mortality from 6.9% in the control group to 35.9% in the over 90 years old patients. Other studies showed the same trend in the increase in mortality in old age [19,20]. This phenomenon can be linked to fewer physiological reserves in the elderly population [21]. Furthemore, a study by Fröhlich et al. from the TraumaRegister DGU^®^ showed that hospitalization of polytrauma survivors is longer in the elderly population, making them more susceptible to nosocomial complications such as multiple organ failure and sepsis, which are also associated with increased mortality [22].

In-hospital mortality was 22.6% in the group with leading head injury, which is higher than 17.2% in the group with more than one leading injury, 6.1% in patients with leading abdominal injury, 4.3% in those with leading thoracic injury, and 3.5% in patients with leading extremity injury. A study comparing the mortality of patients with head injuries and patients without head injuries treated in trauma centers showed a similar mortality rate of 18.2% in patients with head injuries compared to 6.1% in patients without head injuries [23]. A study from Brazil showed traumatic brain injury as an independent early predictor of mortality which is consistent with our data [24].

### 4.3. Risk of Mortality

We were able to show that the risk of mortality increased (17-fold) with age in severely injured patients regardless of the leading injury. This finding confirms age as a dominant and independent prognostic factor in trauma populations. Previous studies showed the same effect. Taylor et al. showed that age is an independent predictor of outcome [25]. Other studies showed an increased mortality in elderly patients [4,20,26,27]. Geriatric patients are thought to be more susceptible to complications due to decreased organ function, less physiological reserves and decreased resistance to infection [28,29]. A case–control study, which analyzed 46,613 major trauma patients and compared 180 elderly major trauma patients (≥65 years) to a similarly injured group of 3918 younger patients, showed a higher mortality linked to complications, especially for pulmonary and infectious complications in geriatric patients with major trauma [21].

In our analysis, however, the magnitude of the age-related increase in mortality differed substantially depending on the leading injury region. While mortality risk increased across all injury types with advancing age, the steepest relative increases were observed in patients with leading abdominal and extremity injuries. In contrast, the increase in mortality risk in patients with leading head injuries was less pronounced in individuals aged over 85 years compared with other anatomical regions. Although in-hospital mortality was greatest in the head injury group at 22.6%, the risk of mortality increased with age by a maximum factor of 11.7. In contrast, the risk of mortality in the 90+ subgroup increased in the entire study population to 17.0, in leading thorax injury to 22.5, in leading extremity injuries to 28.7, and in leading abdomen injury even to 75.2. This could be related to the increasing space in the skull due to brain atrophy, as a study examining brain aging using MRI imaging showed a linear decrease in gray matter over a lifetime, with a marked acceleration in atrophy after the age of 80 [30]. Another possible explanation is that in patients taking anticoagulation drugs, even accidents with relatively low force can result in intracranial bleedings comparable to those seen in younger patients exposed to much higher forces, often accompanied by shearing injuries that may not be detected on a standard CT scan [31,32].

In the context of traumatic head injuries, the observed disparity may potentially originate from the classification provided by the AIS. Since this classification relies on radiological features (especially the extent of the bleeding in cranial CT) and does not account for age or mechanism, injuries of the same severity grade may not be comparable in terms of patient prognosis. Furthermore, it is conceivable that a traumatic head injury caused by high-energy trauma might result in additional damage, such as shearing injuries, which might not always be detectable in a computed tomography scan. Conversely, in the case of elderly patients with coagulopathy due to comorbidities or medications, even a minor trauma can lead to significant bleeding without additional visible injuries. In these two scenarios, it can be assumed that, according to the AIS, the severity of the high-energy trauma is underestimated, while the severity of the low-energy trauma with coagulopathy induced by medication or comorbidity is overestimated. Therefore, the question arises whether uniformly equating intracranial hemorrhages of the same size across different patient cohorts is appropriate. Are intracranial hemorrhages in young and geriatric patients, resulting from high-energy and low-energy trauma, as well as those occurring in individuals with normal coagulation and coagulopathy, equally indicative of injury severity? Or should an adaption be made comparable to the different pelvic fracture classification systems (FFP and Young and Burgess) [33,34]. There are tools that have been developed but are not yet well established, such as the GERtality Score, which combines AIS with physiological conditions and clinical status on admission to improve outcome prediction in older adults [35]. Furthermore, it is imperative to conduct a differential study to ascertain whether reliance solely on computed tomography (CT) scans is sufficient as a diagnostic tool for comprehensive categorization and prognostication. The potential limitations of CT scans in accurately detecting serious injuries, such as shearing injuries, necessitate further investigation.

In contrast, the risk of mortality in old age greatly increases with a leading injury to the extremities and abdomen. This increase in the risk of mortality has not been previously studied in the literature. A possible reason for the increase in mortality due to extremity injuries could be related to more lethal complications in older age [26]. The higher complication rate was shown in a matched-pair analysis which was performed in 2020. The study showed a significantly higher complication rate of 56% in geriatric polytraumatized patients, than in the younger collective with 34% (*p* = 0.031) [36].

### 4.4. Further Predictors

In this analysis, several additional factors were independently associated with mortality across all trauma patients and within subgroups defined by the leading injured body region. A GCS ≤ 8 was the strongest further predictor of death in all groups, with particularly high odds ratios in patients with leading thoracic and abdominal injuries, indicating severe physiological compromise beyond isolated head injury. Multiple studies show that the Glasgow Coma Scale (GCS) has a significant impact on the survival of polytrauma patients [37,38,39]. A study focusing on thoracic injuries (predictors of long-term survival in 5680 patients admitted to a UK major trauma center with thoracic injuries) also showed that a lower Glasgow Coma Scale (GCS) is a predictor of prolonged hospital stay as well as in-hospital mortality [40].

Among these predictors, the New ISS showed a particularly consistent association with mortality. Across all analyses, each additional point in the New ISS increased the odds of death by approximately 5–6%. Although the increase per single ISS point appears numerically small, the cumulative effect becomes clinically highly relevant with increasing trauma severity. For example, a rise of 10 points in the New ISS corresponds approximately to a 60–80% increase in mortality odds. The remarkably narrow confidence intervals further underline the robustness and consistency of the association between injury severity and mortality across all trauma patterns.

Indicators of hemorrhage and physiological derangement, including blood transfusion and coagulopathy, were also associated with increased odds of death across all body regions, with the strongest effect of transfusion observed in thoracic injuries.

Pre-existing comorbidity, reflected by ASA class 3 or 4, showed a moderate but consistent association with mortality, particularly in thoracic and extremity injuries, suggesting reduced physiological reserve contributes to worse outcomes. Male sex demonstrated only a minor association with mortality and was of limited clinical relevance compared with injury severity and physiological factors. Consistent with our findings, the RISC-II score identifies male sex, the presence of a relevant second severe injury (second worst injury, AIS ≥ 3), higher ASA class, and coagulopathy—defined as an international normalized ratio (INR) > 2.4—as independent predictors of mortality in severely injured patients. In addition, RISC-II incorporates blood transfusion as a marker of severe hemorrhage and physiological compromise, reflecting the increased risk of death associated with profound anemia (hemoglobin < 7 g/dL) [41]. Male sex showed only a minor effect across all subgroups.

Overall, these findings indicate that further predictors of death are largely driven by physiological compromise, hemorrhage, and comorbidity, with varying impact depending on the leading injured body region.

### 4.5. Limitations

The present study is a retrospective data analysis based on data of the TraumaRegister DGU^®^. Registry data are less valid than a prospective randomized study due to missing data verification.

Furthermore, it must be noted that many influencing factors can lead to death, in particular, complications during hospitalization. The data analyzed in this study did not allow for a differentiation between the causes of death.

A further limitation relates to the classification of low-height falls. In the present dataset, low falls were defined as falls from a height of less than 3 m according to registry standards. However, this definition is relatively broad and may not adequately capture clinically meaningful distinctions, especially in elderly patients where even ground-level falls can result in severe injury. More granular classification, such as explicit documentation of ground-level or same-height falls, was not consistently available during the study period. This may have introduced heterogeneity within the low-fall category and could influence subgroup comparisons, particularly in the geriatric population.

The very high odds ratios observed, particularly in elderly patients with leading abdominal injuries, should be interpreted with caution. These estimates are likely influenced by the decreasing number of patients with leading abdominal trauma in higher age groups. As the proportion of abdominal injury as the leading injury region declines with age, subgroup sizes become smaller, leading to increased statistical variability and potentially unstable risk estimates. Consequently, the magnitude of association in this subgroup may be overestimated, although the overall trend of increasing mortality with age remains consistent.

Within the context of this scientific publication, the strengths of a multicenter trauma registry study that meticulously collected data from nearly 100,000 patients using a standardized protocol are evident. First and foremost, the inclusion of an extensive patient cohort from multiple centers lends increased external validity to the study and provides insight into a broad range of patient profiles and mechanisms of injury. This increased representativeness supports the transferability of the study findings to the broader population. In addition, the use of a standardized data collection protocol proves to be a key advantage as it ensures consistency of data and minimizes potential bias or data integrity issues, strengthening the robustness of the study’s findings. In addition, the multicenter approach of this study introduces a valuable dimension by including a range of medical institutions. This inclusion not only enriches the diversity of the study’s patient population but also accounts for potential differences in clinical practice and injury patterns in different regions. In essence, these strengths collectively underscore the methodologic rigor and relevance of the multicenter trauma registry study and highlight its contribution to trauma research and patient care.

## 5. Conclusions

With this retrospective cohort study, we were able to show that the risk of mortality increased with age in severely injured patients. Although traumatic head injury had the highest mortality, the increase in risk of mortality with age was less pronounced than in other anatomic regions.

Importantly, our findings suggest that mortality in trauma patients is not determined by injury location alone. Despite comparable injury severity as measured by ISS, elderly patients exhibited substantially higher mortality, indicating that outcome is strongly influenced by patient-related factors such as frailty, comorbidity burden (ASA), and coagulation status rather than anatomical injury patterns alone. From a clinical perspective, these findings highlight that injury severity assessment in elderly trauma patients should not rely solely on anatomical scoring systems. Greater emphasis should be placed on physiological parameters, comorbidity burden, and coagulation status.

Based on these findings, it appears necessary to reevaluate and refine current injury severity assessment tools such as the AIS classification system, particularly in the context of elderly trauma patients and head injuries resulting from low-energy mechanisms.

## Figures and Tables

**Table 1:** Demographics and the characteristics of the study population.

Parameter	Unit	Controls (Age 18–54)	Elderly (Age 55+)
Number of patients	n	44,844	53,637
Sex	males	34,689 (77.4%)	34,567 (64.4%)
Taking anticoagulation drugs	yes	825 (1.8%)	16,672 (31.1%)
Coagulopathy * on admission	yes	3741 (8.3%)	7844 (14.6%)
Pre-trauma condition (ASA)	3/4	1696 (3.8%)	16,928 (31.6%)
Blood transfusion before ICU admission	yes	4343 (9.7%)	3783 (7.1%)
Number of pRBC if transfused	median, Q	4 (2–6)	3 (2–6)
Injury severity score (ISS)	points	20.8 (SD 11.5)	20.4 (SD 10.7)
Maximum AIS = 3		25,777 (57.5%)	28,395 (52.9%)
Maximum AIS = 4		11,597 (25.9%)	14,045 (26.2%)
Maximum AIS = 5/6		7470 (16.6%)	11,197 (20.9%)
Admission to ICU		39,382 (87.8%)	46,946 (87.5%)
Length of stay in ICU	days #	2 (1–7)	3 (1–8)
Length of stay in hospital	days #	12 (6–21)	12 (6–20)
Hospital mortality		3075 (6.9%)	9641 (18.0%)
Leading head injury		7031 (15.7%)	14,622 (27.3%)
Leading thoracic trauma		10,581 (23.6%)	13,214 (24.6%)
Leading abdominal trauma		1700 (3.8%)	742 (1.4%)
Leading extremity injury		7801 (17.4%)	7360 (13.7%)
More than one leading injury		17,731 (39.5%)	17,699 (33.0%)

* Coagulopathy defined as Quick’s value ≤ 60%, or INR ≥ 1.4, or PTT ≥ 40 s; # median and quartiles. PBRC: packed red blood cell, ISS: injury severity score, ASA: American Society of Anesthesiologists risk classification, AIS: Abbreviated Injury, ICU: intensive care unit, n: number of patients.

**Table 2:** Leading injured body region in elderly and control patients, separately for mechanism of injury. Other mechanisms were not included here. The percentages in the rows add up to 100%.

Mechanism of Injury	Groups (Age)	Total (n)	Leading Head (%)	Leading Thorax (%)	Leading Abdomen (%)	Leading Extremities (%)	More than One Leading (%)
Car	18–54	11,106	8.0	27.2	4.5	15.9	44.4
	55+	7559	6.0	43.9	2.9	8.2	39.2
Motorcycle	18–54	8199	4.9	26.8	2.5	27.7	38.0
	55+	4234	4.9	37.3	1.1	19.9	36.8
Bicycle	18–54	4152	28.5	24.7	3.9	10.8	32.0
	55+	5784	28.0	23.8	1.3	9.8	37.2
Pedestrian	18–54	2140	20.1	13.1	1.5	23.0	42.3
	55+	3093	17.6	14.8	0.6	22.7	44.3
High fall (3+ m)	18–54	7650	11.5	16.8	1.1	16.8	53.8
	55+	7592	16.3	25.2	0.5	13.5	44.6
Low fall (<3 m)	18–54	4869	39.1	23.2	2.5	11.4	23.9
	55+	21,321	44.7	17.0	0.8	14.4	23.2
Overall	18–54	44,844	15.7	23.6	3.8	17.4	39.5
	55+	53,637	27.3	24.6	1.4	13.7	33

**Table 3:** In-hospital mortality divided into age groups and leading injured body region (%).

Age Group	n	Mortality					
		Overall	Leading Head	Leading Thorax	Leading Abdomen	Leading Extremities	More than One Leading
18–54	44,844	6.9	9.7	1.9	2.8	0.8	11.8
55–59	9392	7.6	13.1	2.1	4.9	1.1	12.4
60–64	7928	9.2	16.0	2.9	5.8	1.7	13.6
65–69	6699	12.5	21.7	3.5	7.4	3.3	16.6
70–74	6178	15.3	24.5	4.3	16.1	4.2	19.7
75–79	8345	21.0	30.1	8.2	19.6	6.4	26.6
80–84	7573	27.1	36.9	11.3	26.8	10.8	33.2
85–89	4805	34.2	42.8	17.7	37.1	13.1	45.2
90+	2717	35.9	42.0	24.0	61.5	18.6	48.3
**Overall**	**98,481**	**12.9**	**22.6**	**4.3**	**6.1**	**3.5**	**17.2**

**Table 4:** Further predictors for risk of death, expressed as adjusted odds ratios with 95% confidence intervals relative to the control group (age 18–54). Analysis of all cases (overall) was repeated in subgroups of leading body region.

	Overall	Leading Head	Leading Thorax	Leading Extremities	More than One Leading
Male sex	1.1 (1.1–1.2)	1.1 (1.0–1.2)	1.2 (1.0–1.5)	1.3 (1.1–1.6)	1.1 (1.0–1.2)
New ISS (per point)	1.05 (1.05–1.05)	1.05 (1.04–1.05)	1.05 (1.05–1.06)	1.06 (1.05–1.08)	1.06 (1.06–1.06)
GCS ≤ 8	7.3 (6.9–7.7)	7.4 (6.7–8.2)	17.0 (14.0–20.9)	6.2 (4.3–8.9)	5.2 (4.8–5.6)
ASA 3/4	1.7 (1.6–1.8)	1.3 (1.2–1.5)	2.9 (2.5–3.5)	3.1 (2.4–3.8)	1.7 (1.5–1.8)
Blood transfusion	2.2 (2.1–2.4)	2.5 (2.1–3.1)	6.9 (5.3–9.0)	2.5 (1.9–3.4)	1.9 (1.7–2.0)
Coagulopathy	2.5 (2.4–2.7)	2.6 (2.4–2.9)	2.3 (2.0–2.8)	2.2 (1.7–2.7)	2.6 (2.4–2.8)

## Data Availability

The datasets are available from the corresponding author on reasonable request.

## Abbreviations

The following abbreviations are used in this manuscript:

A	Austria
AIS	Abbreviated Injury Scale
ASA	American Society of Anesthesiologists (risk classification)
AUC	Academy for Trauma Surgery (Akademie der Unfallchirurgie GmbH)
CH	Switzerland
CT	Computed Tomography
D	Germany
DGU	Deutsche Gesellschaft für Unfallchirurgie (German Society for Trauma Surgery)
FFP	Fragility Fractures of the Pelvis
GCS	Glasgow Coma Scale
ICM	Intensive Care Medicine
ICU	Intensive Care Unit
IFOM	Institute for Research in Operative Medicine
INR	International Normalized Ratio
ISS	Injury Severity Score
MRI	Magnetic Resonance Imaging
NIS	Notfall-, Intensiv- und Schwerverletztenmedizin (Emergency, intensive care and severely injured medicine)
OR	Odds Ratio
PBRC	Packed Red Blood Cells
PTT	Partial Thromboplastin Time
RISC-II	Revised Injury Severity Classification, Version II
SD	Standard Deviation
STROBE	Strengthening the Reporting of Observational Studies in Epidemiology
TR-DGU	TraumaRegister DGU^®^
WHO	World Health Organization

## References

[1] World Health Organization (WHO) (2025). Ageing and Health.

[2] Bundesamt für Statistik (BFS) (2024). Bevölkerung: Stand und Entwicklung.

[3] European Commission, Eurostat (2021). Demography of Europe—2021 Edition.

[4] Giannoudis P., Harwood P., Court-Brown C., Pape H. (2009). Severe and multiple trauma in older patients; incidence and mortality. Injury.

[5] Wutzler S., Lefering R., Laurer H.L., Walcher F., Wyen H., Marzi I. (2008). Changes in geriatric traumatology. An analysis of 14,869 patients from the German Trauma Registry. Unfallchirurg.

[6] van der Sluis C.K., Klasen H., Eisma W., Ten Duis H. (1996). Major trauma in young and old: What is the difference?. J. Trauma Acute Care Surg..

[7] Brown C.V., Rix K., Klein A.L., Ford B., Teixeira P.G., Aydelotte J., Coopwood B., Ali S. (2016). A comprehensive investigation of comorbidities, mechanisms, injury patterns, and outcomes in geriatric blunt trauma patients. Am. Surg..

[8] Eaton J., Grudziak J., Hanif A.B., Chisenga W.C., Hadar E., Charles A. (2017). The effect of anatomic location of injury on mortality risk in a resource-poor setting. Injury.

[9] Spering C., Lefering R., Bouillon B., Lehmann W., von Eckardstein K., Dresing K., Sehmisch S. (2020). It is time for a change in the management of elderly severely injured patients! An analysis of 126,015 patients from the TraumaRegister DGU®. Eur. J. Trauma Emerg. Surg..

[10] Shamliyan T., Talley K.M., Ramakrishnan R., Kane R.L. (2013). Association of frailty with survival: A systematic literature review. Ageing Res. Rev..

[11] Sterling D.A., O’connor J.A., Bonadies J. (2001). Geriatric falls: Injury severity is high and disproportionate to mechanism. J. Trauma Acute Care Surg..

[12] Clegg A., Young J., Iliffe S., Rikkert M.O., Rockwood K. (2013). Frailty in elderly people. Lancet.

[13] Peck K.A., Calvo R.Y., Schechter M.S., Sise C.B., Kahl J.E., Shackford M.C., Shackford S.R., Sise M.J., Blaskiewicz D.J. (2014). The impact of preinjury anticoagulants and prescription antiplatelet agents on outcomes in older patients with traumatic brain injury. J. Trauma Acute Care Surg..

[14] AUC—Akademie der Unfallchirurgie GmbH (2025). TraumaRegister DGU®.

[15] Von Elm E., Altman D.G., Egger M., Pocock S.J., Gøtzsche P.C., Vandenbroucke J.P. (2007). The Strengthening the Reporting of Observational Studies in Epidemiology (STROBE) statement: Guidelines for reporting observational studies. Lancet.

[16] Association for the Advancement of Automotive Medicine (AAAM) (2025). Abbreviated Injury Scale (AIS).

[17] Osler T., Baker S.P., Long W. (1997). A modification of the injury severity score that both improves accuracy and simplifies scoring. J. Trauma Acute Care Surg..

[18] Rauer T., Aschwanden A., Rothrauff B.B., Pape H.-C., Scherer J. (2023). Fractures of the lower extremity after e-bike, bicycle, and motorcycle accidents: A retrospective cohort study of 624 patients. Int. J. Environ. Res. Public Health.

[19] Ziegenhain F., Scherer J., Kalbas Y., Neuhaus V., Lefering R., Teuben M., Sprengel K., Pape H.-C., Jensen K.O., Dgu T. (2021). Age-dependent patient and trauma characteristics and hospital resource requirements—Can improvement be made? An analysis from the German Trauma Registry. Medicina.

[20] de Vries R., Reininga I.H., de Graaf M.W., Heineman E., El Moumni M., Wendt K.W. (2019). Older polytrauma: Mortality and complications. Injury.

[21] Finelli F.C., Jonsson J., Champion H.R., Morelli S., Fouty W.J. (1989). A case control study for major trauma in geriatric patients. J. Trauma Acute Care Surg..

[22] Fröhlich M., Lefering R., Probst C., Paffrath T., Schneider M.M., Maegele M., Sakka S.G., Bouillon B., Wafaisade A. (2014). Epidemiology and risk factors of multiple-organ failure after multiple trauma: An analysis of 31,154 patients from the TraumaRegister DGU. J. Trauma Acute Care Surg..

[23] Gennarelli T.A., Champion H.R., Sacco W.J., Copes W.S., Alves W.M. (1989). Mortality of patients with head injury and extracranial injury treated in trauma centers. J. Trauma Acute Care Surg..

[24] da Costa L.G.V., Carmona M.J.C., Malbouisson L.M., Rizoli S., Rocha-Filho J.A., Cardoso R.G., Auler-Junior J.O.C. (2017). Independent early predictors of mortality in polytrauma patients: A prospective, observational, longitudinal study. Clinics.

[25] Taylor M.D., Tracy J.K., Meyer W., Pasquale M., Napolitano L.M. (2002). Trauma in the elderly: Intensive care unit resource use and outcome. J. Trauma Acute Care Surg..

[26] Clement N.D., Tennant C., Muwanga C. (2010). Polytrauma in the elderly: Predictors of the cause and time of death. Scand. J. Trauma Resusc. Emerg. Med..

[27] Aldrian S., Nau T., Koenig F., Vécsei V. (2005). Geriatric polytrauma. Wien. Klin. Wochenschr..

[28] King D.W., Pushparaj N., O’Toole K. (1982). Morbidity and mortality in the aged. Hosp. Pract..

[29] Oreskovich M., Howard J., Copass M., Carrico C. (1985). Geriatric Trauma: Injury Patterns and Outcome. Surv. Anesthesiol..

[30] Coupé P., Catheline G., Lanuza E., Manjón J.V., Initiative A.s.D.N. (2017). Towards a unified analysis of brain maturation and aging across the entire lifespan: A MRI analysis. Hum. Brain Mapp..

[31] Davis A.E. (2000). Mechanisms of traumatic brain injury: Biomechanical, structural and cellular considerations. Crit. Care Nurs. Q..

[32] Kim J.J., Gean A.D. (2011). Imaging for the diagnosis and management of traumatic brain injury. Neurotherapeutics.

[33] Rommens P.M., Hofmann A. (2013). Comprehensive classification of fragility fractures of the pelvic ring: Recommendations for surgical treatment. Injury.

[34] Burgess A.R., Eastridge B.J., Young J.W., Ellison T.S., Ellison P.S., Poka A., Bathon G.H., Brumback R.J. (1990). Pelvic ring disruptions: Effective classification system and treatment protocols. J. Trauma Acute Care Surg..

[35] Scherer J., Kalbas Y., Ziegenhain F., Neuhaus V., Lefering R., Teuben M., Sprengel K., Pape H.-C., Jensen K.O. (2021). The GERtality score: The development of a simple tool to help predict in-hospital mortality in geriatric trauma patients. J. Clin. Med..

[36] Jensen K.O., Lempert M., Sprengel K., Simmen H.P., Pothmann C., Schlögl M., Bischoff-Ferrari H.A., Hierholzer C., Pape H.C., Neuhaus V. (2020). Is there any difference in the outcome of geriatric and non-geriatric severely injured patients?—A seven-year, retrospective, observational cohort study with matched-pair analysis. J. Clin. Med..

[37] Vorbeck J., Bachmann M., Düsing H., Hartensuer R. (2023). Mortality risk factors of severely injured polytrauma patients (prehospital mortality prediction score). J. Clin. Med..

[38] Emircan Ş., Özgüç H., Aydın Ş.A., Özdemir F., Köksal Ö., Bulut M. (2011). Factors affecting mortality in patients with thorax trauma. Ulus. Travma Acil Cerrahi Derg..

[39] Rupprecht H., Heppner H.J., Wohlfart K., Türkoğlu A. (2017). The geriatric polytrauma: Risk profile and prognostic factors. Turk. J. Trauma Emerg. Surg..

[40] Ariyaratnam P., Lee A., Milton R., Troxler M., Barlow I., Ferrier G., Scott D. (2023). Predictors of long-term survival in 5,680 patients admitted to a UK major trauma centre with thoracic injuries. Ann. R. Coll. Surg. Engl..

[41] Lefering R., Huber-Wagner S., Nienaber U., Maegele M., Bouillon B. (2014). Update of the trauma risk adjustment model of the TraumaRegister DGU™: The Revised Injury Severity Classification, version II. Crit. Care.

